# Parents’ coping with their adolescent’s negative emotions following internet-delivered emotion regulation therapy for adolescents with non-suicidal self-injury disorder: a secondary analysis of a randomised clinical trial

**DOI:** 10.1136/bmjment-2025-302039

**Published:** 2026-01-29

**Authors:** Olivia Ojala, Katja Sjöblom, Hugo Hesser, Erik Hedman-Lagerlöf, Clara Hellner, Johan Bjureberg

**Affiliations:** 1 Centre for Psychiatry Research, Department of Clinical Neuroscience, Karolinska Institutet & Region Stockholm, Stockholm, Sweden; 2 School of Behavioural, Social and Legal Sciences, Örebro University, Örebro, Sweden; 3 Department of Clinical Neuroscience, Division of Psychology, Karolinska Institutet, Stockholm, Sweden

**Keywords:** Psychotherapy, Mental Health Teletherapy, Emotion-Focused Therapy, Behavior Therapy

## Abstract

**Background:**

Non-suicidal self-injury (NSSI) among adolescents is linked to adverse outcomes for youth and their families. While parental involvement is considered essential in treating adolescent NSSI, the effects on parents remain unclear.

**Objective:**

To evaluate if Internet-delivered Emotion Regulation Individual Therapy for Adolescents (IERITA) adjunctive to treatment as usual (TAU) is superior to TAU alone in improving parental coping with adolescents’ negative emotions, the durability of potential effects and whether reductions in parents’ minimising of their adolescent’s emotions mediate adolescent outcomes.

**Methods:**

166 adolescents with NSSI disorder (93% female; mean (SD) age=15.0 (1.2) years) and their parents (83% female; mean (SD) age=46.5 (5.1) years) were randomised to IERITA plus TAU (n=84) or TAU-only (n=82). IERITA is a 12-week, therapist-guided emotion regulation behavioural therapy, including both adolescents and parents. In parallel to the adolescent, parents participate in a separate internet-delivered course designed to provide skills for understanding and supporting their adolescent. The outcomes were parental coping measured by the Coping with Children’s Negative Emotions Scale-Adolescent version, including minimisation (primary outcome), distress, punitive and expressive encouragement responses at baseline, post-treatment (primary endpoint) and 3 months post-treatment. Parental minimisation was tested as a mediator of adolescent NSSI absence and emotion regulation difficulties.

**Findings:**

There were statistically significant treatment effects on parental minimisation and distress responses at post-treatment and 3 months post-treatment, and punitive responses at 3 months post-treatment, favouring IERITA. Parental minimisation did not mediate adolescent NSSI absence or emotion regulation difficulties.

**Conclusion:**

IERITA shows promise for supporting parents of youth with NSSI. Further studies are needed to understand how parental improvement may impact youth with NSSI.

**Clinical implications:**

Online family treatment for NSSI can improve how parents handle their adolescents’ emotions.

**Trial registration number:**

NCT03353961.

**WHAT IS ALREADY KNOWN ON THIS TOPIC**

**WHAT THIS STUDY ADDS**

- This study showed that a 12-week online intervention, targeting both adolescents engaging in NSSI and their parents, can contribute to improvements in parents’ coping with their adolescent’s negative emotions. In addition, parents’ minimisation of their adolescent’s emotions did not mediate adolescent NSSI absence and emotion regulation difficulties.

**HOW THIS STUDY MIGHT AFFECT RESEARCH, PRACTICE OR POLICY**

- These results highlight the importance of parent-focused approaches in the clinical practice and treatment of adolescent NSSI and further studies are needed to better understand how potential parental improvement may impact adolescents with NSSI.

## Background

 Non-suicidal self-injury (NSSI) among youth is a global health concern, with an estimated prevalence of 17%.^1^ NSSI involves the intentional harm to one’s body tissue without suicidal intent and for reasons that are neither socially nor culturally sanctioned.^2^ NSSI is associated with various mental health problems, including recurrent self-harm, alcohol misuse^3^ and suicide attempts.^4^ In addition, youth NSSI can significantly strain family dynamics and adversely impact parental well-being, leading to increased distress and higher rates of work absence.^5 6^ In turn, youth who engage in NSSI often describe their relationships with parents as emotionally distant, marked by a lack of perceived protection and elevated parental control.^7^ Parental invalidation (eg, judging, rejecting or minimising their adolescent’s emotions) is theorised to play a central role in both the development and maintenance of self-injury.^8^ Linehan’s biosocial theory posits that self-injury develops and is maintained as a result of difficulties in regulating emotions, a capacity formed by the interaction between an individual’s biological predisposition to emotional reactivity and an invalidating environment.^8^ Indeed, evidence suggests that controlling parental styles, low degree of parental support and high reactive control from parents are associated with greater NSSI risk among youth.^9 10^ In addition, higher parental invalidation has predicted less favourable treatment outcomes regarding adolescent NSSI frequency.^11^ Investigating parental invalidation as a potential mechanism of change may inform more effective interventions for adolescent self-injury.

Involving parents and enhancing family functioning is crucial in treating adolescent NSSI.^12 13^ We have developed and, in several studies, evaluated a 12-week Internet-delivered Emotion Regulation Individual Therapy for Adolescents (IERITA) with NSSI and their parents.^14^,^16^ In IERITA, parents enrol in a therapist-supported course, focusing on supporting their adolescent in effective emotion regulation and communication. A recent randomised controlled trial (RCT) showed that IERITA, adjunctive to treatment as usual (TAU), was superior to TAU only, for several adolescent outcomes, including NSSI frequency.^14^ However, the question remains whether and how parents benefit from the IERITA parent course.

Few treatment studies for adolescents with NSSI have evaluated the effects on parent coping and communication and have yielded mixed findings. A small RCT (n=38) found that neither dialectical behaviour therapy for adolescents (DBT-A) nor supportive therapy predicted change in parental validation or invalidation.^17^ However, an uncontrolled study on DBT-A indicated that DBT-A was associated with improvements in parent–child communication.^18^ Moreover, a feasibility study of IERITA (n=25)^15^ showed that parents’ punitive and minimisation responses to adolescents’ negative emotions decreased and that expressive encouragement responses improved significantly from pretreatment to post-treatment. Thus, the treatment effect on parental response styles has not yet been evaluated in an adequately powered RCT.

## Objective

This study constitutes a secondary analysis from a recent RCT of IERITA.^14^ The first aim of the current study was to investigate the effects of IERITA on parents’ coping with adolescents’ negative emotions. We expected that IERITA plus TAU, relative to TAU only, would result in larger reductions in parents’ minimising (primary outcome), punitive, distress and expressive encouragement responses to their adolescent’s emotions. The second aim was to explore the durability of the effects, expecting durability at 3 months post-treatment. The third aim was to study mediation, expecting that effects on parents’ minimisation would mediate the proportion of NSSI absence and effects in emotion regulation difficulties 1 month post-treatment.

## Methods

### Design

In total, 166 dyads, consisting of one adolescent and one parent, were randomised to either IERITA plus TAU (n=84) or TAU only (n=82). Study sites in Sweden included the child and adolescent psychiatry clinics in Stockholm, Västra Götaland and Skåne. Parents and adolescents provided written informed consent (if the adolescent was ≤14 years old; parents provided consent on their behalf). The study has been registered at Clinicaltrials.gov (Identifier NCT03353961). Data collection started in November 2017 and ended in January 2021. Detailed descriptions of methods and the full trial protocol are available elsewhere^14^ and in the EMethods in the online supplemental file 1 (ie, power calculation and adverse events). We focus here on the relevant procedures, measures and interventions to this report.

### Participant recruitment, selection, allocation and setting

Participants were recruited through clinician referrals or self-referral. Pretreatment assessments included telephone screening and a face-to-face assessment with a clinical psychologist or psychotherapist. Inclusion criteria included adolescents aged 13–17 years who met the diagnostic criteria for NSSI disorder in accordance with The Diagnostic and Statistical Manual of Mental Disorders, Fifth Edition, had engaged in ≥1 NSSI episode in the past month, and had at least one parent willing to participate in the parent course. Exclusion criteria for adolescents were: (1) immediate suicide risk; (2) diagnosis of psychotic, bipolar I disorder or current (past month) substance dependence; (3) other primary mental disorder requiring immediate and different treatment (eg, severe anorexia nervosa); (4) insufficient understanding of the Swedish language; (5) life circumstances that could interfere with or prevent treatment participation, or that required immediate intervention (eg, violence in close relationships); and (6) a clinician assessed global functioning level of Children’s Global Assessment Scale^19^ of <40.^14^ The random allocation sequence was generated using a true random number service (generating randomness via atmospheric noise) and administered by an independent researcher in blocks of four or six per treatment clinic and placed in opaque sealed envelopes. Families were randomised (1:1) without stratification to treatment allocation.

### Procedures

Parental outcomes were self-reported by parents at pretreatment, post-treatment (primary endpoint) and 3 months post-treatment. In the case of two parents, only one was permitted to complete the self-report measures. The family determined which parent would participate, and the same parent provided responses at all assessments.

Adolescent outcome of past month NSSI was measured by assessor at baseline, and masked assessors by telephone at 1 month post-treatment. Adolescent outcome of emotion regulation difficulties was measured through online self-reports by adolescents at baseline and 1 month post-treatment. At all time points, adolescent emotion regulation difficulties were based on weekly responses over 4 weeks (ie, week −3 to 0 prior to treatment initiation (pretreatment) and week 13 to 16 (1 month post-treatment)). The total score at each time point was calculated as an average across those 4 weeks.

### Interventions

#### Internet-delivered Emotion Regulation Individual Therapy for Adolescents

IERITA is an acceptance-based behavioural intervention developed from emotion regulation group therapy, DBT and acceptance and commitment therapy, and optimised through several pilot studies.^12 15 16^ The goal and suggested treatment mechanism in IERITA is to improve adolescents’ emotion regulation skills to reduce NSSI. The intervention constitutes an adolescent treatment offered in eleven modules across 12 weeks and a separate parent course.^14^

The IERITA parent course aims to teach parents skills to tackle their emotional reactions to having a child engaging in NSSI and provide skills for understanding and supporting their adolescents. The parent course is structured into six modules on a secure digital platform, which can be completed over either 6 or 12 weeks, based on the parent’s preference. The parent has access to the material covered in the adolescent’s treatment. Parents (and adolescents) are given weekly homework assignments and have regular support from an online therapist (psychologist or psychotherapist) on the platform. The parent course consists of psychoeducation about NSSI, related emotional states and the practice of emotional awareness. Parents are also taught to recognise and practise understanding and acceptance of their own and others’ emotional experiences (eg, validation). In addition, behavioural activation is introduced, encouraging the parent to spend one-on-one time with their adolescent as well as time alone for recovery. The treatment concludes with a summary and guidance on managing challenges and setbacks, reinforcing knowledge and sustaining the benefits achieved. See online supplemental table S1 for an overview of the content presented in each module. Parents completed on average 5.5 (SD=1.0) modules out of 6, and therapists spent on average 378.8 (SD=163.1) minutes per family in total on reviewing and providing feedback.^14^

#### Treatment as usual

All participants were given treatment recommendations or referral before randomisation. Families in both conditions were allowed TAU according to their needs. TAU was offered at community clinics by clinicians employed at the clinics (ie, not study personnel). Participants could have different types of TAU (psychotherapy, psychotropics), frequency and focus (eg, focusing on comorbidity). Hence, what constituted TAU was different for different participants. The most common counselling type in both conditions was supportive therapy, offered every second week or once a month. This amounted to four to eight sessions from pretreatment to 1 month post-treatment. See online supplemental table S2 for additional characteristics of TAU in both conditions. Type and amount of TAU did not differ between groups.^14^

### Outcomes

#### Parental outcomes

Parents’ perceived ability to handle and respond to their adolescent’s negative emotions was evaluated using the Coping with Children’s Negative Emotions Scale-Adolescent version (CCNES-A).^20^ The CCNES-A presents parents with nine hypothetical scenarios that depict typical situations likely to evoke teenagers’ negative emotions (eg, *‘*When I see my teenager become anxious about something at school, I usually…’). Parents are asked to rate the likelihood of using different responses to their adolescent’s negative emotions on a seven-point Likert-type scale. The parental responses and subscales assessed for each scenario investigated in this study were: (1) *minimisation* response, which measures the degree to which the parent discounts or invalidates their adolescent’s negative emotion (eg, *‘*I tell him/her that he/she is making too big a deal out of it’); (2) *distress* responses, which measures to what degree the parent may become aroused or distressed themselves by their adolescent’s negative emotions (e*g, ‘*I become nervous and uneasy in dealing with his/her anxiety’); (3) *punitive response*, which measures to what degree the parent uses punishment aiming at controlling their adolescent’s negative emotion (eg, *‘*I get angry at him/her for not dealing with things better’) and lastly; (4) *expressive encouragement* responses, which measures the degree to which the parent actively encourages their adolescent’s expression of negative emotions (eg, *‘*I encourage him/her to talk about what is making him/her nervous’). These four subscales (9 items each; 36 items in total) were selected a priori based on the theorised core target of the IERITA parental course (ie, increasing emotional acceptance and reducing invalidation and emotional unwillingness) and findings indicating that controlling parental behaviours specifically are associated with increased NSSI frequency.^9 10^ The total subscale score ranges from 1 to 7 and the mean is presented as the average response. Lower scores indicate more desired response, except for the subscale expressive encouragement, for which higher scores indicate more desired response.^20^ CCNES exhibits good test–retest reliability and internal consistency: minimisation (α=0.78), distress (α=0.70), punitive (α=0.69) and expressive encouragement responses (α=0.85)^21^

#### Adolescent outcomes

Adolescent emotion regulation difficulties were measured with The Difficulties in Emotion Regulation Scale (DERS-16),^22^ a 16-item measure of emotion regulation difficulties. DERS-16 measures five different emotion regulation difficulties: (1) lack of emotional clarity (two items); (2) difficulties engaging in goal-directed behaviour (three items); (3) difficulties with impulse control (three items); (4) limited access to effective emotion regulation strategies (five items); (5) non-acceptance of emotional responses (three items). Adolescents were asked to indicate, on a five-point Likert-type scale, how often each statement regarding difficulties in emotion regulation applied to them. Scores on this measure range from 16 to 80, with higher scores indicating greater difficulties.^22^

DERS-16 has demonstrated good reliability and validity among adolescents.^23^

NSSI was measured by a clinician using the Deliberate Self Harm Inventory-Youth version (DSHI-Y).^24^ The DSHI-Y is a youth-adapted version of the Deliberate Self-Harm Inventory,^24^ with adequate construct, convergent and discriminant validity, and test–retest reliability.^24^ This measure consists of six items and evaluates the frequency of the most common forms of NSSI, such as cutting, burning, severe scratching, self-biting, self-punching and head banging.^24^ The absence of NSSI was defined as having no NSSI during the past 30 days and was measured as a binary variable (yes/no).

### Statistical analyses

Treatment effects (total, direct and indirect) were evaluated using linear and logistic regression analysis for continuous and binary variables within the structural equation modelling framework (ie, path analysis).^25^ Univariate regression models were first conducted to evaluate overall effects for parents on all variables at post-treatment (aim 1) and 3 months post-treatment (aim 2) assessments by including the treatment variable as a binary predictor (IERITA plus TAU=1, TAU=0). Models with continuous outcome variables covaried pretreatment levels on the same variable measured at baseline, and continuous predictors were grand mean centred (similar to analysis of covariance). Based on the model-implied effects, effect sizes were calculated as standardised mean difference (Cohen’s *d*) and OR for continuous and binary outcomes, respectively. However, for within-group effect sizes, Cohen’s *d* was calculated using the mean difference and the average SD from both measures.

Mediation was evaluated using counterfactually defined causal indirect and direct effects (aim 3). Counterfactually defined effects provide a general approach to mediation, extending mediation to non-linear models (eg, binary mediator or outcome) and accounting for mediator-treatment interaction effects.^26^ Online supplemental figure S2 depicts a schematic mediation model. As shown in the figure, the mediator (minimisation responses) at post-treatment (week 12) and the outcome (adolescent NSSI absence (week 16) and emotion regulation difficulties (pooled value for week 13–16), respectively) at 1 month follow-up were each analysed in separate models, with both outcomes regressed on the binary treatment variable along with any pretreatment covariate. In addition, the outcome is regressed on the mediator as well as the interaction between mediator and treatment variable. Indirect and direct effects (ie, total natural indirect effect and pure natural direct effect) were significance tested using asymmetric CIs based on bootstrapping (5000 samples drawn with replacements). Two separate mediation models were conducted to estimate the mediated effect of minimisation responses (measured with CCNES-A) for each of the two adolescent outcomes: NSSI absence (measured with DSHI-Y) and emotion regulation difficulties (measured with DERS-16). If evidence was provided for mediation, the following analyses were planned: (1) analyses to assess the robustness of the results to violations of the assumption of no confounding of the mediator–outcome relationship, (2) observed pretreatment variables that may serve as confounders were to be included, and were models reestimated and (3) sensitivity analysis for unmeasured pretreatment confounders following recommendations.^27^

All models were fitted with full information maximum likelihood estimation and non-normality robust SEs (MLR; returned by MLR or bootstrapping) using Mplus versus 8.1.^28^ Graphs were constructed using MplusAutomation, semPlot and ggplot2 packages in R (references in online supplemental file 1). Throughout, comparisons were treated as statistically significant at p<0.05 (two-tailed). Models included all randomised participants who had at least one observation on the dependent variable. Parameter estimates and SEs were jointly estimated using all available data, with missing data handled under the missing at-random assumption. To test the robustness of results to the handling of missing data, sensitivity analyses were conducted using multiple imputation (EMethods in online supplemental file 1).

## Findings

**Figure 1:**
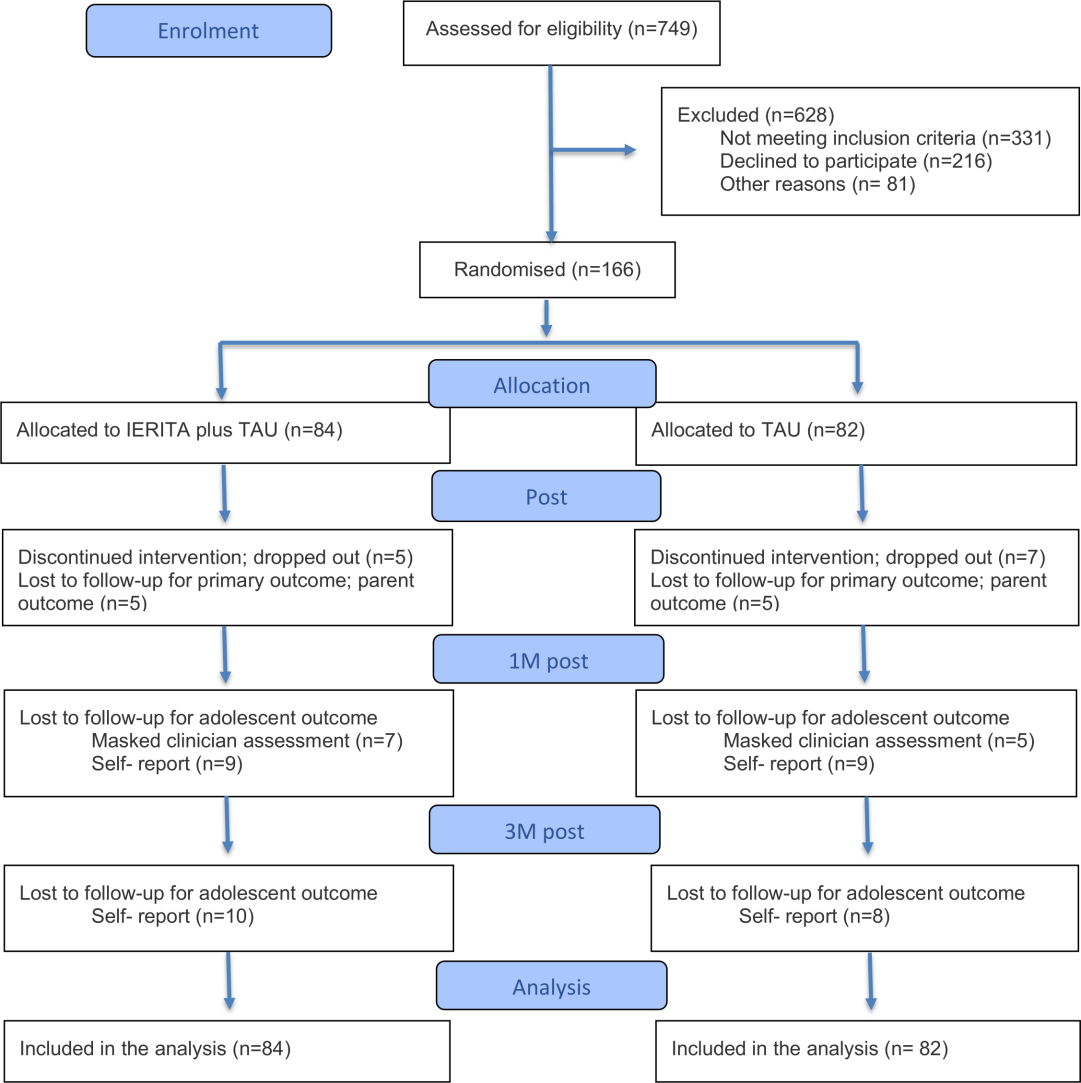
Flow diagram of patient enrolment and disposition. IERITA, Internet-delivered Emotion Regulation Individual Therapy for Adolescents; 1M, one-month post-treatment; TAU, treatment as usual.

A total of 166 adolescent and parent dyads were recruited and randomised to IERITA plus TAU (84 participants) or TAU only (82 participants). 12 dyads (7%; 5 in IERITA plus TAU, 7 in TAU only) dropped out of the treatment. Families in which the parent did not complete the CCNES-A at post-treatment (primary endpoint) did not differ on any baseline characteristics from parents who completed the measurement (online supplemental table S3). In addition, and as reported elsewhere, missingness in adolescent data was not strongly related to baseline characteristics.^14^ Study, parent participant and youth participant characteristics are presented in table 1. A participant flow diagram is presented in figure 1.

**Table 1:** Study, parent and youth characteristics

	No. (%)	No. (%)	No. (%)
	IERITA+TAU (n=84)	TAU (n=82)	Total (n=166)
Study characteristics			
Source of referral			
Clinician	55 (65)	46 (56)	101 (61)
Self	29 (35)	36 (44)	65 (39)
Parent characteristics			
Gender			
Female	68 (81)	69 (84)	137 (83)
Male	16 (19)	13 (16)	29 (17)
Relation to child participant			
Biological mother	66 (79)	66 (80)	132 (80)
Biological father	16 (19)	12 (15)	28 (17)
Adoptive parent	1 (1)	2 (2)	3 (2)
Other	1 (1)	2 (2)	3 (2)
Age, mean (SD)	46.79 (5.13)	46.15 (5.07)	46.47 (5.10)
Region of birth			
Sweden	76 (90)	78 (95)	154 (93)
Asia/South or North America/Europe	8 (10)	4 (5)	12 (7)
Biological children			
1	10 (13)	13 (17)	23 (15)
2	39 (49)	33 (43)	72 (46)
3	23 (29)	26 (34)	49 (31)
≥4	8 (10)	5 (6)	13 (8)
Parent living arrangement			
With children	67 (80)	67 (82)	134 (81)
With spouse/partner	58 (70)	63 (77)	121 (73)
Alone	4 (5)	2 (2)	6 (4)
Parent education level			
Primary school	1 (1)	2 (2)	3 (2)
Secondary school	35 (42)	31 (38)	66 (40)
College/university <3 years	7 (8)	8 (10)	15 (9)
College/university ≥3 years	35 (42)	37 (45)	72 (43)
Doctorate	6 (7)	4 (5)	10 (6)
Parent occupational status			
Employed or self-employed	82 (98)	77 (94)	159 (96)
Unemployed/sick leave/retired	2 (2)	5 (6)	7 (4)
Parent clinical characteristics			
Life-time NSSI, yes	13 (16)	10 (12)	23 (14)
Life-time suicide attempt, yes	6 (7)	2 (2)	8 (5)
Life-time mental disorder, yes*	27 (32)	33 (40)	60 (36)
Youth characteristics			
Gender			
Female	77 (92)	77 (94)	154 (93)
Male	5 (6)	2 (2)	7 (4)
Non-binary	2 (2)	3 (4)	5 (3)
Age, mean (SD)	15.04 (1.31)	15.02 (1.19)	15.03 (1.25)
Region of birth			
Sweden	80 (95)	80 (98)	160 (96)
Asia/South or North America/Europe	4 (5)	2 (2)	6 (4)
Youth clinical characteristics			
Age NSSI onset, mean (SD),	12.70 (1.27)	12.51 (1.57)	12.61 (1.42)
Years since NSSI onset, mean (SD)	2.33 (1.36)	2.51 (1.31)	2.42 (1.33)
Comorbidity†			
Major depressive disorder	49 (58)	48 (59)	97 (58)
Anxiety disorders			
Social anxiety disorder	24 (29)	23 (28)	47 (28)
Panic disorder/agoraphobia	17 (20)	11 (13)	28 (17)
Specific phobia disorder	14 (17)	13 (16)	27 (16)
Generalised anxiety disorder	12 (14)	9 (11)	21 (13)
ADHD‡	14 (17)	15 (18)	29 (18)
Autism spectrum disorder	4 (5)	3 (4)	7 (4)
OCD/BDD	3 (4)	7 (9)	10 (6)
Eating disorder§	6 (7)	1 (1)	7 (4)
Oppositional defiant disorder	3 (4)	2 (2)	5 (3)
Mean (SD) number of co-occurring disorders	1.93 (1.76)	1.85 (1.52)	1.89 (1.64)
Mean (SD) number of BPD criteria¶	1.88 (1.28)	2.11 (1.54)	1.99 (1.42)
Fulfilling ≥5 BPD criteria	5 (6)	7 (9)	12 (7.2)
Suicidality			
Low	37 (44)	37 (45)	74 (45)
Moderate	21 (25)	21 (26)	42 (25)
High	26 (31)	24 (29)	50 (30)
Life-time suicide attempt, yes**	13 (16)	12 (15)	25 (15)
Ever received inpatient care, yes	2 (2)	2 (2)	4 (2)
Previous counselling, yes	54 (64)	53 (65)	107 (64)
Number of months in previous counselling, mean (SD)	10.6 (13.8)	10.2 (14.8)	10.4 (14.3)
Any ongoing psychopharmacological medication, yes	31 (37)	25 (31)	56 (34)

* Self-reported answer to the question ‘Have you ever been diagnosed with a mental disorder within health care services?’

† Assessed by the research team using the MINI-KID (Mini-International Neuropsychiatric Interview for Children and Adolescents), V.6 and the Body Dysmorphic Disorder Questionnaire (administered as an interview).

‡ Includes both combined, primarily inattentive and primarily hyperactive–impulsive subtype.

§ Includes anorexia nervosa and bulimia nervosa.

¶ Assessed by the research team using the Structured Clinical Interview for DSM.

** In total, eight participants (4.8%) had missing value on this variable.

ADHD, attention-deficit hyperactivity disorder; BDD, body dysmorphic disorder; BPD, borderline personality disorder; DSM, Diagnostic and Statistical Manual of Mental Disorders; IERITA, Internet-delivered Emotion Regulation Individual Therapy for Adolescents; NSSI, nonsuicidal self-injury; OCD, obsessive-compulsive disorder; TAU, treatment as usual.

### Effects on parental outcomes

Table 2 presents unstandardised regression coefficients for the linear regressions on the treatment variable, with associated between-group standardised effect sizes (ie, Cohen’s *d*), for all variables at 1 month and 3 month post-treatment. Online supplemental table S4 presents within-group effect sizes. In terms of aim 1, there were statistically significant treatment effects on all variables at post-treatment with effect sizes in the small to moderate range (minimisation response; *d*: 0.37, 95% CI 0.17 to 0.57, distress response; *d*: 0.25, 95% CI 0.05 to 0.45), favouring IERITA in all cases, except for punitive responses and expressive encouragement responses, where no statistically significant effects were observed. Results were consistent with the primary analyses when using multiple imputation (online supplemental table S5).

**Table 2:** Descriptive statistics on outcomes and results from linear regression analysis

	IERITA+TAU		TAU		Fixed effects		Effect size	
	Mean (SD)	N (%)	Mean (SD)	N (%)	β (SE)	P value	*d*	95% CI
Minimisation response								
Pretreatment	2.90 (1.03)	84 (100)	2.63 (0.98)	82 (100)				
Post-treatment	1.96 (0.81)	79 (94)	2.18 (0.85)	76 (93)	−0.38 (0.10)	0.001	0.37	0.17 to 0.57
3M post-treatment	1.99 (0.89)	74 (88)	2.22 (0.89)	74 (90)	−0.40 (0.12)	<0.001	0.40	0.17 to 0.62
Distress response								
Pretreatment	1.89 (0.94)	84 (100)	1.77 (0.68)	82 (100)				
Post-treatment	1.48 (0.55)	79 (94)	1.65 (0.69)	76 (93)	−0.20 (0.09)	0.017	0.25	0.05 to 0.45
3M post-treatment	1.46 (0.61)	74 (88)	1.72 (0.72)	74 (90)	−0.27 (0.10)	0.006	0.33	0.10 to 0.57
Punitive response								
Pretreatment	1.51 (0.59)	84 (100)	1.46 (0.46)	82 (100)				
Post-treatment	1.29 (0.42)	79 (94)	1.29 (0.42)	76 (93)	−0.09 (0.05)	0.109	0.16	−0.04 to 0.36
3M post-treatment	1.29 (0.45)	74 (88)	1.44 (0.43)	74 (90)	−0.16 (0.06)	0.004	0.32	0.10 to 0.53
Expressive encouragement response								
Pretreatment	5.22 (0.93)	84 (100)	5.19 (0.97)	82 (100)				
Post-treatment	5.58 (0.80)	79 (94)	5.36 (0.91)	76 (93)	0.19 (0.11)	0.089	0.20	−0.03 to 0.44
3M post-treatment	5.55 (0.92)	74 (88)	5.28 (0.9)	74 (90)	0.25 (0.13)	0.056	0.26	−0.01 to 0.52

*d*, Cohen’s *d*; IERITA, Internet-delivered Emotion Regulation Individual Therapy for Adolescents; 3M, 3 months; TAU, treatment as usual.

### Durability of the effects on parental outcomes

In terms of aim 2, all statistically significant effects observed at post-treatment were also significant at 3 months post-treatment (minimisation response; *d*: 0.40, 95% post-treatment CI 0.17 to 0.62, distress response; *d*: 0.33, 95% CI 0.10 to 0.57. Additionally, statistically significant effects were observed in punitive responses at 3 months post-treatment (punitive responses; *d*: 0.32, 95% CI 0.10 to 0.53 favouring IERITA). Lastly, there were no statistically significant effects observed in expressive encouragement responses at 3 months post-treatment. Findings remained consistent under multiple imputation (online supplemental table S5).

### Mediation models

Online supplemental table S6 presents unstandardised regression coefficients for the evaluated mediation models, and online supplemental table S7 presents the bootstrapped CIs (90%, 95% and 99%) for the counterfactually defined indirect and direct effects. In line with what has already been reported elsewhere,^14^ treatment had a statistically significant effect on NSSI absence and emotion dysregulation, run in separate models. Treatment also had a statistically significant effect on the mediator (minimisation responses). The mediator had no main effect on any of the outcomes (adolescent NSSI absence or emotion regulation difficulties), nor was the mediator by treatment interaction effect statistically significant. Thus, none of the mediated effects were statistically significantly different from zero as evaluated with bootstrapped asymmetric CIs (online supplemental figure S1). Results from post hoc baseline cross-sectional analyses including mediator and outcomes are presented in the EResults (in online supplemental file 1).

## Discussion

The findings from this RCT support the efficacy of IERITA delivered adjunctively to TAU in improving parents’ response to their adolescent’s negative emotions. IERITA plus TAU, compared with TAU alone, resulted in statistically significantly greater reduction in minimisation (a form of invalidation) and distress responses at both post-treatment and 3 month post-treatment, suggesting durability of effects. IERITA plus TAU also reduced punitive responses more, relative to TAU, at 3 month post-treatment. However, there were no significant differences in expressive encouragement, and minimisation did not mediate adolescent NSSI absence or emotion regulation difficulties. These findings extend prior research by demonstrating that a relatively brief and resource-efficient intervention can significantly influence parental responses, thereby contributing to meaningful improvements in parent–adolescent interactions.

The strongest treatment effects were found for parents’ minimisation responses, in line with the previous IERITA feasibility trial.^15^ Validation strategies are a central component of the IERITA parent course, and our findings align with previous qualitative research highlighting validation as particularly valuable from the parents’ perspective.^12^ The results indicate that parental invalidation can decrease through targeted clinical intervention. Moreover, providing treatment materials and dedicated therapist contact for parents, as implemented in IERITA, may be important for reducing parental invalidation. As expected, parents in the IERITA plus TAU group reported less distress responses (eg, anxious, uncomfortable and nervous) and fewer punitive responses (eg, anger, threats, scolding) to their adolescent’s negative emotions compared with TAU alone. The IERITA parent course’s components, such as psychoeducation on emotions, self-practice in emotional awareness and self-validation, may have contributed to these outcomes. These findings are clinically relevant, as reduced parental distress has been linked to better coping with setbacks and modelling of adaptive emotion regulation.^12 29^ Further, it is possible that the treatment effects generalised to other family members, including siblings or other parents. Future research should explore potential intervention effects across the broader family system.

In contrast to our hypothesis, we did not find significant treatment effects in expressive encouragement (ie, encouraging adolescents to express their emotions). A potential explanation for this finding is that we observed high ratings at baseline, meaning that parents already demonstrated high levels of expressive encouragement pretreatment and there was limited room for improvement. In fact, similar potential floor effects may help explain the effect sizes observed for the non-supportive strategies (ie, distress, punitive and minimisation; Cohen’s *d*=0.16–0.40). Nevertheless, small effects may still be meaningful in a trial of this kind, particularly with an active comparator.

Parents’ minimisation of the adolescent’s emotions at post-treatment did not mediate changes in adolescent NSSI absence or emotion regulation difficulties at 1 month post-treatment (ie, b-path was not significant). This may reflect a delayed impact of reduced parental invalidation on adolescent outcomes or the influence of other, more salient mediators. To note, reduced adolescent emotion regulation difficulties were shown to mediate a reduction in NSSI frequency in the main report of the IERITA RCT.^14^ Thus, targeting adolescent emotion regulation in IERITA remains the most effective approach to reduce NSSI. While this study does not clarify how parental changes contribute to adolescent outcomes, adolescents may still benefit from reduced parental invalidation in ways not captured here. Evidence indicates that parental involvement can improve youth outcomes, including reduced anxiety, and may benefit parental well-being, although this remains largely unexplored.^30^ Qualitative data suggest enhanced parent–child relationships post-treatment, including more open communication and fewer conflicts.^12^ Future research should explore additional mediators and outcomes to refine the role of parental involvement in interventions for adolescent NSSI. One promising mediator is parental emotion regulation difficulties based on previous findings supporting adolescent emotion regulating difficulties as a mediator in IERITA,^14^ and given that emotion regulation is the primary treatment target of both the adolescent and parent components. Lastly, it is important to note that a reverse causal pathway is possible; for example, an adolescent’s improvement may mediate parental improvement in coping with the adolescent’s negative emotions. Given that both adolescents and parents have shown improvements following IERITA, it is possible that each influenced the other. Exploring this further could provide a broader understanding of change processes within the family.

### Strengths and limitations

A strength of this RCT is the evaluation of treatment effects in both adolescents and parents, providing a broad understanding of how IERITA plus TAU affects the adolescent–parent dyad. Additionally, missing data were minimal. The 3 months post-treatment period enables evaluations of the stability of the effects of the intervention over time. Future studies of IERITA should preferably include longer follow-ups to understand long-term durability of treatment effects.

The study also has limitations. First, it exclusively relies on unblinded parental assessments of their own behaviour using one scale, omitting the adolescent’s perspective. The augmentation design, adding a parent course to one group, may have primed the IERITA parents to understand and want to report helpful parent coping, thus potentially inflating the reported improvements. Hence, social desirability and expectancy effects may have impacted the validity of the findings negatively. Importantly, however, previous research has demonstrated that the CCNES is not meaningfully associated with social desirability (except for distress responses)^21^ and fully blinding participants is difficult in psychological treatment trials. Thus, this study should be seen as a first step in understanding parental change within the context of treatment for adolescent NSSI. Future research should incorporate adolescents’ perspectives on their parents’ behaviour (eg, using the CCNES—adolescent perception version) along with blinded clinician observations to reduce bias and provide a more nuanced and comprehensive understanding of the parental changes. Second, although the augmentation design is appropriate for early-stage treatment development, future studies should compare IERITA with similar parent-involved interventions to clarify its specific effects. Third, the predominately female parent sample limits the generalisability of our findings. Subsequent IERITA studies should strive for greater gender diversity, facilitating investigation of effects across genders. Finally, although comparison with TAU enhances ecological validity, unknown variability in parental involvement within TAU limits the interpretability of the findings. Future research should determine the most effective ways to combine IERITA and TAU to best support diverse families.

## Clinical implications

Augmenting TAU with IERITA appears to enhance several key aspects of parental coping with adolescents’ negative emotions, relative to TAU alone. This scalable 12-week online intervention, comprising six parental modules, shows promise for supporting parents of youth engaging in NSSI and may reach families who face barriers to traditional treatment due to time or geography. Further research is warranted to examine additional outcomes and mediators to clarify how parental improvements may contribute to adolescent treatment response.

## Supplementary material

10.1136/bmjment-2025-302039 online supplemental file 1

## Data Availability

No data are available.

## References

[1] Moloney F, Amini J, Sinyor M (2024). Sex Differences in the Global Prevalence of Nonsuicidal Self-Injury in Adolescents: A Meta-Analysis. JAMA Netw Open.

[2] The International Society for the Study of Self-Injury (2025). About self-injury [internet]. https://www.itriples.org/aboutnssi.

[3] Bjureberg J, Ohlis A, Ljótsson B (2019). Adolescent self-harm with and without suicidality: cross-sectional and longitudinal analyses of a Swedish regional register. *J Child Psychol Psychiatry*.

[4] Ohlis A, Bjureberg J, Lichtenstein P (2020). Comparison of suicide risk and other outcomes among boys and girls who self-harm. Eur Child Adolesc Psychiatry.

[5] Arbuthnott AE, Lewis SP (2015). Parents of youth who self-injure: a review of the literature and implications for mental health professionals. Child Adolesc Psychiatry Ment Health.

[6] Karemyr M, Gubi E, Ohlis A (2025). Work absence in parents of youth who self-harm. *BMJ Ment Health*.

[7] Bureau JF, Martin J, Freynet N (2010). Perceived Dimensions of Parenting and Non-suicidal Self-injury in Young Adults. *J Youth Adolescence*.

[8] Linehan MM (1993). Cognitive-Behavioral Treatment of Borderline Personality Disorder.

[9] Fong ZH, Loh WNC, Fong YJ (2022). Parenting behaviors, parenting styles, and non-suicidal self-injury in young people: a systematic review. Clin Child Psychol Psychiatry.

[10] Baetens I, Claes L, Onghena P (2015). The effects of nonsuicidal self-injury on parenting behaviors: a longitudinal analyses of the perspective of the parent. Child Adolesc Psychiatry Ment Health.

[11] Ojala O, Hesser H, Gratz KL (2024). Moderators and predictors of treatment outcome following adjunctive internet-delivered emotion regulation therapy relative to treatment as usual alone for adolescents with nonsuicidal self-injury disorder: Randomized controlled trial. *JCPP Adv*.

[12] Simonsson O, Engberg H, Bjureberg J (2021). Experiences of an Online Treatment for Adolescents With Nonsuicidal Self-injury and Their Caregivers: Qualitative Study. *JMIR Form Res*.

[13] Johansson BA, Wilbe Ramsay K, Pettersson A (2025). Effects of interventions for self-harm in children and adolescents: a systematic review and meta-analysis. Eur Child Adolesc Psychiatry.

[14] Bjureberg J, Ojala O, Hesser H (2023). Effect of Internet-Delivered Emotion Regulation Individual Therapy for Adolescents With Nonsuicidal Self-Injury Disorder: A Randomized Clinical Trial. JAMA Netw Open.

[15] Bjureberg J, Sahlin H, Hedman-Lagerlöf E (2018). Extending research on Emotion Regulation Individual Therapy for Adolescents (ERITA) with nonsuicidal self-injury disorder: open pilot trial and mediation analysis of a novel online version. BMC Psychiatry.

[16] Morthorst B, Olsen MH, Jakobsen JC (2022). Internet based intervention (Emotion Regulation Individual Therapy for Adolescents) as add-on to treatment as usual versus treatment as usual for non-suicidal self-injury in adolescent outpatients: The TEENS randomised feasibility trial. *JCPP Adv*.

[17] Adrian M, Berk MS, Korslund K (2018). Parental Validation and Invalidation Predict Adolescent Self-Harm. Prof Psychol Res Pr.

[18] Smith L, Hunt K, Parker S (2023). Parent and Carer Skills Groups in Dialectical Behaviour Therapy for High-Risk Adolescents with Severe Emotion Dysregulation: A Mixed-Methods Evaluation of Participants’ Outcomes and Experiences. *IJERPH*.

[19] Shaffer D, Gould MS, Brasic J (1983). A children’s global assessment scale (CGAS). Arch Gen Psychiatry.

[20] Fabes RA, Eisenberg N, Bernzweig J (1990). Coping with Children’s Negative Emotions Scale (CCNES): Description and Scoring.

[21] Fabes RA, Poulin RE, Eisenberg N (2002). Department of Family & Human Development. Marriage Fam Rev.

[22] Bjureberg J, Ljótsson B, Tull MT (2016). Development and Validation of a Brief Version of the Difficulties in Emotion Regulation Scale: The DERS-16. J Psychopathol Behav Assess.

[23] Monell E, Birgegård A, Nordgren L (2022). Factor structure and clinical correlates of the original and 16-item version of the Difficulties In Emotion Regulation Scale in adolescent girls with eating disorders. J Clin Psychol.

[24] Gratz KL (2001). Measurement of Deliberate Self-Harm: Preliminary Data on the Deliberate Self-Harm Inventory. J Psychopathol Behav Assess.

[25] Muthén BO, Muthén LK, Asparouhov T (2017). Regression and Mediation Analysis Using Mplus.

[26] Valeri L, Vanderweele TJ (2013). Mediation analysis allowing for exposure-mediator interactions and causal interpretation: theoretical assumptions and implementation with SAS and SPSS macros. Psychol Methods.

[27] Imai K, Keele L, Tingley D (2010). A general approach to causal mediation analysis. Psychol Methods.

[28] Muthén M (2017). Mplus User’s Guide [Internet].

[29] Flynn D, Gillespie C, Joyce M (2023). An evaluation of the skills group component of DBT-A for parent/guardians: a mixed methods study. Ir J Psychol Med.

[30] Lawrence PJ, Parkinson M, Jasper B (2021). Supporting the parents of children and young people with anxiety and depressive disorders is an opportunity not to be missed: a scoping review. Lancet Psychiatry.

