# Supplementary material for: Parents’ coping with their adolescent’s negative emotions following internet-delivered emotion regulation therapy for adolescents with non-suicidal self-injury disorder: a secondary analysis of a randomised clinical trial
Source: BMJ Ment Health. 2026 Jan 29;29(1):e302039. doi: 10.1136/bmjment-2025-302039 (PMC12863314; doi:10.1136/bmjment-2025-302039)
Supplement: online supplemental file 1 [file bmjment-29-1-s001.docx]

Supplementary material for “Parents' Coping with Their Adolescent's Negative Emotions Following Internet-Delivered Emotion Regulation Therapy for Adolescents with Nonsuicidal Self-Injury Disorder: A Secondary Analysis of a Randomized Clinical Trial” by Ojala et al

Contents

[EMethods 2](#_Toc218502340)

[EResults 3](#_Toc218502341)

[Figure S1 4](#_Toc218502342)

[Figure S2 5](#_Toc218502343)

[Table S1 6](#_Toc218502344)

[Table S2 8](#_Toc218502345)

[Table S3 10](#_Toc218502346)

[Table S4 13](#_Toc218502347)

[Table S5 14](#_Toc218502348)

[Table S6 15](#_Toc218502349)

[Table S7 16](#_Toc218502350)

[References 17](#_Toc218502351)

# EMethods

**Power**

Power was calculated for the primary report of the IERITA trial.^1^ Statistical power was estimated using a 0.05-level Wald test for the interaction between a binary treatment variable and linear time in treatment (12 weeks) in a zero-inflated negative binomial regression model using 200 bootstrap samples. Based on a previous open trial of IERITA,^2^ an average difference of two NSSI episodes between the two conditions at 1-month post-treatment was expected. Given this, the power analysis revealed a sample size of 166 participants and parents with a power of 82%, alpha=.05, and a maximum attrition rate of 15%.

**Adverse events**

As reported in the primary report,^1^ adverse events were measured among participating adolescents. In total, 16 out of 80 (20.0%) adolescents responding to the adverse events scale in the IERITA plus TAU group reported experiencing negative effects during the treatment period. Of these, 4 (5.0%) adverse events were related to the treatment at post-treatment. Adverse events among adolescents included experiencing increased sadness, stress and self-destructive behaviours. Five adolescents (6%) allocated to IERITA plus TAU reported suicide attempts, compared to 8 adolescents (10%) in the TAU only condition during the 12-week treatment period. At 3-months post-treatment, one participant reported a suicide attempt in the TAU only condition. One participant in the IERITA plus TAU condition was reported dead by accident at 3-months post-treatment. In Sweden all suspected suicide attempts are investigated. These investigations encompass all healthcare providers involved in the care of the deceased. The research team was not contacted as part of such an investigation.

**Statistical analysis**

Graphs were constructed using MplusAutomation,^3^ semPlot,^4^ and ggplot2^5^ packages in R.^6^ Sensitivity analyses using multiple imputation were carried out to examine whether the method of handling missing data affected the results. All outcome models were re-estimated using multiple imputation under the missing at-random assumption. A total of 50 imputed data sets were generated in Mplus (at every 500th imputation cycle). The imputation model included treatment group and all outcome variables included in the respective analyses. Regression analyses were conducted in Mplus using TYPE = IMPUTATION, with parameter estimates and standard errors combined across imputations using Rubin’s^7^ pooling equations.

# EResults

An exploratory post-hoc analysis to understand the link between mediator and outcomes at baseline showed small and non-significant correlations (NSSI: *rho =* 0.002, 95% CI –0.15, 0.15, p = 0.976; emotion regulation difficulties: *r =* 0.048*,* 95% CI –0.11, 0.20, p = 0.542).

# Figure S1

Bootstrap distribution of the pure natural indirect effect for outcome a) NSSI Absence, b) Emotion regulation difficulties. The dotted vertical lines represent 95% upper and lower bound.

# Figure S2

Schematic figure of the mediation model


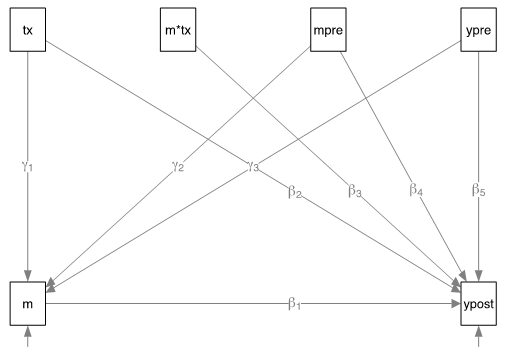


*Note.* This figure depicts a schematic mediation model. The mediator (minimization responses) at post-treatment (week 12) and the outcome (adolescent NSSI absence [week 16] and emotion regulation difficulties [pooled value for week 13-16], respectively) at one-month follow-up were each analyzed in separate models, with both outcomes regressed on the binary treatment variable along with any pre-treatment covariate. In addition, the outcome is regressed on the mediator as well as the interaction between mediator and treatment variable.

m = mediator at post-treatment; mpre = mediator at pre-treatment; m*tx = mediator by treatment interaction; tx = treatment variable; ypre = outcome at pre-treatment; ypost = outcome at one-month post-treatment

# Table S1

Content of the Parent course in Internet-delivered Emotion Regulation Individual Therapy for Adolescents

| **Module** | **Theme** | **Content** |
| --- | --- | --- |
| **Module 1** | **Psychoeducation** | - Information about how internet-delivered treatment works and advice on how to structure the treatment work - Psychoeducation about NSSI and emotional reactivity   *Homework*: Observe emotional reactivity and its consequences in yourself and others. |
| **Module 2** | **Emotional awareness** | - Psychoeducation about emotions: what they are, their components (thoughts, bodily sensations, and action urges), why we have them, and common emotions and how they can be experienced - The difference between describing and judging emotions   *Homework*: Practice emotional awareness |
| **Module 3** | **Validation and invalidation** | - Psychoeducation about validation and invalidation - Learn about contextual factors that can contribute to an invalidating environment (e.g., not noticing the emotions that are expressed, not knowing what to do) - Notice situations where one has been invalidating - Learn that validation can be a way to regulate emotions and have a positive impact on relationships - Practice self-validation - Examples of how one can validate others - Half-time evaluation of how the treatment work is going. Investigate additional need for support   *Homework:* Practice validation of others; try three different ways to validate. |
| **Module 4** | **Self-validation and self-invalidation** | - Common pitfalls with validation and how to handle them - Psychoeducation on self-invalidation, the contexts and situations in which it can occur, and what emotions it can lead to - Learn and practice self-validation.   *Homework:* (1) Continue practicing validation of others. (2) Practice self-validation once a day. |
| **Module 5** | **Behavioral activation** | - Learn about behavioral activation for yourself - Learn about behavioral activation together with your adolescent - Strategies to ask the adolescent to engage in an activity together - Suggestions of activities to do together to get more positive time together   *Homework*: (1) Engage in activities that are enjoyable or relaxing (2) Engage in activities together with the adolescent on the adolescent’s terms. |
| **Module 6** | **Summary** | - Summary and follow-up - Plan for continued practice of skills - Evaluation of what has been helpful - Possibility to download all the material |

*Note*. Previously described in Bjureberg, J., Ojala, O., Hesser, H., Häbel, H., Sahlin, H., Gratz, K. L., Tull, M. T., Claesdotter Knutsson, E., Hedman-Lagerlöf, E., Ljótsson, B., & Hellner, C. (2023). Effect of Internet-Delivered Emotion Regulation Individual Therapy for Adolescents With Nonsuicidal Self-Injury Disorder: A Randomized Clinical Trial. *JAMA Network Open*, *6*(7), e2322069. https://doi.org/10.1001/jamanetworkopen.2023.22069

# Table S2

Characteristics of Treatment as Usual

| Characteristics | Participants, n (%) | |
| --- | --- | --- |
| **Baseline to 1-month post-treatment** | | |
|  | IERITA plus TAU  (n = 79) | TAU only  (n = 76) |
| Received counselling | 62 (78.5) | 56 (73.7) |
| Counselling type |  |  |
| Supportive therapy | 48 (77.4) | 47 (83.9) |
| Cognitive behavior therapy | 6 (9.7) | 5 (8.9) |
| Do not know | 8 (10.1) | 4 (5.2) |
| Frequency of counselling |  |  |
| Every week | 11 (17.8) | 13 (23.2) |
| Every second week | 18 (29.0) | 20 (35.7) |
| Once per month | 23 (37.1) | 14 (25.0) |
| Less than once per month | 9 (14.5) | 9 (16.1) |
| Never | 1 (1.6) | 0 |
| Inpatient care | 0 | 1 (1.3) |
| Any ongoing psychopharmacological medication | 32 (46) | 35 (41) |
| **1-month post-treatment to 3-months post-treatment^a^** | | |
|  | IERITA plus TAU^a^  (n = 74) | TAU only  (n = 74) |
| Received counselling | 48 (64.9) | 52 (70.3) |
| Counselling type |  |  |
| Supportive therapy | 35 (72.9) | 37 (71.2) |
| Cognitive behavior therapy | 7 (14.6) | 11 (21.2) |
| Do not know | 6 (8.1) | 2 (2.7) |
| Frequency of counselling |  |  |
| Every week | 7 (14.6) | 12 (23.1) |
| Every second week | 11 (22.9) | 13 (25.0) |
| Once per month | 16 (33.3) | 14 (26.9) |
| Less than once per month | 14 (29.2) | 13 (25.0) |
| Never | 0 | 0 |
| Inpatient care | 0 | 1 (1.4) |
| Any ongoing psychopharmacological medication | 35 (46) | 31 (42) |
| *Note*. Previously reported in Bjureberg, J., Ojala, O., Hesser, H., Häbel, H., Sahlin, H., Gratz, K. L., Tull, M. T., Claesdotter Knutsson, E., Hedman-Lagerlöf, E., Ljótsson, B., & Hellner, C. (2023). Effect of Internet-Delivered Emotion Regulation Individual Therapy for Adolescents With Nonsuicidal Self-Injury Disorder: A Randomized Clinical Trial. *JAMA Network Open*, *6*(7), e2322069. https://doi.org/10.1001/jamanetworkopen.2023.22069  ^a^Pharmacological results included 76 participants in the IERITA plus TAU group.  Abbreviations: IERITA, internet-delivered emotion regulation therapy for adolescents; TAU, treatment as usual. | | |

# Table S3

Study, Parent, and Youth Characteristics of Participants with Complete

Data and Missing Data on Coping with Children's Negative Emotions Scale - Adolescent version at Post-treatment (Primary Endpoint)

|  | **Complete data** | **Missing data** | **Chi2/Fischer’s exact test/T** | ***P*** |
| --- | --- | --- | --- | --- |
| No (%) | **155 (93)** | **11 (7)** |  |  |
| **Study Characteristics** |  |  |  |  |
| Source of referral |  |  | 1.772 | 0.531 |
| Clinician | 93 (60) | 8 (73) |  |  |
| Self | 62 (40) | 3 (27) |  |  |
| **Parent Characteristics** |  |  |  |  |
| Gender |  |  | 1.053 | 1.000 |
| Female | 128 (83) | 9 (82) |  |  |
| Male | 27 (17) | 2 (18) |  |  |
| Relation to Child Participant |  |  | 0.446 | 0.931 |
| Biological mother | 123 (79) | 9 (82) |  |  |
| Biological father | 26 (17) | 2 (18) |  |  |
| Adoptive parent | 3 (2) | 0 (0) |  |  |
| Other | 3 (2) | 0 (0) |  |  |
| Age, mean (SD) | 46.45 (5.05) | 46.91 (5.86) | -0.256 | 0.803 |
| Region of birth |  |  | 0.765 | 0.574 |
| Sweden | 144 (93) | 10 (91) |  |  |
| Asia/South or North America/Europe | 11 (7) | 1 (9) |  |  |
| Biological children |  |  | 0.948 | 0.814 |
| 1 | 21(14) | 2 (18) |  |  |
| 2 | 66 (45) | 6 (55) |  |  |
| 3 | 47 (32) | 2 (18) |  |  |
| ≥4 | 12 (8) | 1 (9) |  |  |
| Parent living arrangement |  |  |  |  |
| With children | 126 (82) | 8 (73) | 0.616 | 0.445 |
| With spouse/partner | 113 (73) | 8 (73) | 0.991 | 1.000 |
| Alone | 6 (4) | 0 (0) | <0.001 | 1.000 |
| Parent education level |  |  |  |  |
| Primary school | 3 (2) | 0 (0) | 1.704 | 0.790 |
| Secondary school | 60 (39) | 6 (54) |  |  |
| College/university < 3 years | 14 (9) | 1 (9) |  |  |
| College/university ≥ 3 years | 68 (44) | 4 (36) |  |  |
| Doctorate | 10 (6) | 0 (0) |  |  |
| Parent occupational status |  |  | 0.154 | 0.070 |
| Employed or self-employed | 150 (97) | 9 (82) |  |  |
| Unemployed/sick leave/retired | 5 (3) | 2 (18) |  |  |
| **Parent Clinical Characteristics** |  |  |  |  |
| Life-time NSSI, yes | 23 (15) | 0 (0) | 0.879 | 0.349 |
| Life-time suicide attempt, yes | 6 (4) | 2 (18) | 1.949 | 0.163 |
| Life-time psychiatric disorder, yes^a^ | 54 (35) | 6 (55) | 0.980 | 0.322 |
| Table S3. Study, Parent, and Youth Characteristics of Participants with Complete  Data and Missing Data on Coping with Children's Negative Emotions Scale - Adolescent version at Post-treatment (Primary Endpoint) (Continued) | | | | |
|  | **Complete data** | **Missing data** | **Chi2/Fischer’s exact test/T** | ***P*** |
| **Youth Characteristics** |  |  |  |  |
| Gender |  |  | 0.918 | 0.632 |
| Female | 143 (92) | 11 (100) |  |  |
| Male | 7 (5) | 0 (0) |  |  |
| Non-binary | 5 (3) | 0 (0) |  |  |
| Age, mean (SD) | 15.05 (1.23) | 14.75 (1.51) | 0.655 | 0.526 |
| Region of birth |  |  | <0.001 | 1.000 |
| Sweden | 149 (96) | 11 (100) |  |  |
| Asia/South or North    America/Europe | 6 (4) | 0 (0) |  |  |
| **Youth Clinical Characteristics** |  |  |  |  |
| Age NSSI onset, mean (SD), | 12.65 (1.42) | 12.00 (1.34) | 1.550 | 0.148 |
| Years since NSSI onset, mean (SD) | 2.40 (1.34) | 2.75 (1.24) | -0.894 | 0.389 |
| Comorbidity^b^ |  |  |  |  |
| Major depressive disorder | 89 (57) | 8 (72) | 1.970 | 0.364 |
| Anxiety disorders |  |  |  |  |
| Social anxiety disorder | 44 (28) | 3 (27) | 0.946 | 1.000 |
| Panic disorder/Agoraphobia | 27 (17) | 1 (9) | 0.476 | 0.692 |
| Specific phobia disorder | 26 (17) | 1 (9) | 0.498 | 1.000 |
| Generalized anxiety disorder | 19 (12) | 2 (18) | 1.585 | 0.633 |
| ADHD^c^ | 28 (18) | 1 (9) | 0.455 | 0.691 |
| Autism spectrum disorder | 6 (4) | 1 (9) | 2.464 | 0.387 |
| OCD/BDD | 9 (6) | 1 (9) | 1.617 | 0.506 |
| Eating disorder^d^ | 7 (5) | 0 (0) | <0.001 | 1.000 |
| Oppositional defiant disorder | 5 (3) | 0 (0) | <0.001 | 1.000 |
| Mean (SD) number of co-occurring disorders | 1.82 (1.54) | 1.82 (1.66) | 0.002 | 0.998 |
| Mean (SD) number of BPD criteria^e^ | 1.98 (1.42) | 2.18 (1.40) | -0.460 | 0.654 |
| Fulfilling ≥5 BPD criteria | 11 (7) | 1 (9) | 1.307 | 0.574 |
| Suicidality |  |  | 0.838 | 0.658 |
| Low | 68 (44) | 6 (55) |  |  |
| Moderate | 39 (25) | 3 (27) |  |  |
| High | 48 (31) | 2 (18) |  |  |
| Life-time suicide attempt, yes^f^ | 23 (16) | 2 (18) | 1.197 | 0.686 |
| Ever received inpatient care, yes | 4 (3) | 0 (0) | <0.001 | 1.000 |
| Previous counselling, yes | 99 (64) | 8 (73) | 1.504 | 0.748 |
| Number of months in previous counselling, mean (SD) | 10.2 (14.3) | 16.1 (15.0) | -1.156 | 0.278 |
| Any ongoing psychopharmacological medication, yes | 51 (33) | 5 (45) | 0.271 | 0.603 |

*Note.* Chi-square or Fischer’s exact test were used for categorical variables and T-test was used for continuous variables. Fischer’s exact test was used if one cell had 1-4 observations and in 2x2 tables.

Abbreviations: ADHD, attention-deficit hyperactivity disorder; BDD, body dysmorphic disorder; BPD,

borderline personality disorder IERITA, internet-delivered emotion regulation individual therapy; NSSI, nonsuicidal self-injury; OCD, obsessive-compulsive disorder SD, standard deviation; TAU, treatment as usual

^a^Self-reported answer to the question “Have you ever been diagnosed with a psychiatric disorder within health care services”?

^b^ Assessed by the research team using the MINI-KID International Neuropsychiatric Interview, version 6 and the Body Dysmorphic Disorder Questionnaire (administered as an interview).

^c^ Includes both combined, primarily inattentive, and primarily hyperactive-impulsive subtype.

^d^ Includes anorexia nervosa and bulimia nervosa.

^e^Assessed by the research team using the Structured Clinical Interview for DSM.

^f^ In total 8 participants (4.8%) had missing value on this variable

# Table S4

Within-Group Effects from Observed Values on the Coping with Children's Negative Emotions Scale- Adolescent version

*Note.* Cohen’s *d* was calculated using the mean difference between the measures and the average standard deviation from both measures.

Abbreviations: FU, Follow-up (three months); IERITA, Internet-delivered Emotion Regulation Individual Therapy; TAU, Treatment As Usual; *d,* Cohen's *d*

| Group | Comparison | Effect size | |
| --- | --- | --- | --- |
|  |  | *d* | 95% CI |
| **Minimization response** | | | |
| IERITA + TAU | Pre-Post | 1.02 | 0.76, 1.29 |
| IERITA + TAU | Pre-FU | 0.95 | 0.69, 1.21 |
| TAU | Pre-Post | 0.49 | 0.26, 0.72 |
| TAU | Pre-Post | 0.44 | 0.22, 0.67 |
| **Distress response** | | | |
| IERITA + TAU | Pre-Post | 0.54 | 0.31, 0.77 |
| IERITA + TAU | Pre-FU | 0.55 | 0.32, 0.78 |
| TAU | Pre-Post | 0.17 | -0.05, 0.39 |
| TAU | Pre-Post | 0.07 | -0.15, 0.29 |
| **Punitive response** | | | |
| IERITA + TAU | Pre-Post | 0.43 | 0.21, 0.66 |
| IERITA + TAU | Pre-FU | 0.42 | 0.20, 0.65 |
| TAU | Pre-Post | 0.24 | 0.02, 0.46 |
| TAU | Pre-Post | 0.05 | -0.17, 0.26 |
| **Expressive encouragement response** | | | |
| IERITA + TAU | Pre-Post | 0.42 | 0.19, 0.64 |
| IERITA + TAU | Pre-FU | 0.35 | 0.13, 0.57 |
| TAU | Pre-Post | 0.18 | -0.04, 0.40 |
| TAU | Pre-Post | 0.09 | -0.13, 0.31 |

# Table S5

Results from regression analysis using multiple imputation

|  | Time | β (SE) | *P* | Effect size (*d*) | 95% CI |
| --- | --- | --- | --- | --- | --- |
| **Minimization response** | | | | | |
|  | Post-treatment | -0.37 (0.10) | <.001 | 0.37 | 0.17, 0.57 |
|  | 3M post-treatment | -0.40 (0.11) | <.001 | 0.40 | 0.18, 0.62 |
| **Distress response** | | | | | |
|  | Post-treatment | -0.20 (0.08) | 0.017 | 0.24 | 0.04, 0.45 |
|  | 3M post-treatment | -0.27 (0.10) | 0.005 | 0.33 | 0.10, 0.56 |
| **Punitive response** | | | | | |
|  | Post-treatment | -0.08 (0.05) | 0.127 | 0.16 | -0.04, 0.36 |
|  | 3M post-treatment | -0.17 (0.06) | 0.003 | 0.33 | 0.12, 0.54 |
| **Expressive encouragement response** | | | | | |
|  | Post-treatment | 0.19 (0.11) | 0.094 | 0.20 | -0.03, 0.43 |
|  | 3M post-treatment | 0.25 (0.13) | 0.055 | 0.26 | -0.01, 0.52 |

Abbreviations: 3M, three-month; SE, standard error; d, Cohen's d

# Table S6

Unstandardized estimates from mediation models

| Variable | Estimate | SE | *p* | LowerCI | UpperCI |
| --- | --- | --- | --- | --- | --- |
| **NSSI Absence at 1-month post-treatment** |  |  |  |  |  |
| Minimization response post-treatment <- Minimization response pre-treatment | 0.50 | 0.06 | <0.001 | 0.38 | 0.62 |
| Minimization response post-treatment <- Treatment | -0.38 | 0.11 | <0.001 | -0.58 | -0.17 |
| NSSI absence 1-month post-treatment <- Minimization response pre-treatment | 0.25 | 0.23 | 0.272 | -0.15 | 0.75 |
| NSSI absence 1-month post-treatment <- Minimization response post-treatment | 0.10 | 0.34 | 0.770 | -0.58 | 0.74 |
| NSSI absence 1-month post-treatment <- Minimization response post-treatment X Treatment | -0.51 | 0.50 | 0.305 | -1.64 | 0.32 |
| NSSI absence 1-month post-treatment <- Treatment | -0.96 | 0.39 | 0.013 | -1.80 | -0.30 |
| **Emotion regulation difficulties at 1-month post-treatment** |  |  |  |  |  |
| Minimization response post-treatment <- Minimization response pre-treatment | 0.50 | 0.06 | <0.001 | 0.38 | 0.63 |
| Minimization response post-treatment <- Emotion regulation difficulties pre-treatment | -0.00 | 0.01 | 0.583 | -0.01 | 0.01 |
| Minimization response post-treatment <- Treatment | -0.40 | 0.10 | <0.001 | -0.58 | -0.18 |
| Emotion regulation difficulties 1-month post-treatment <- Minimization response pre-treatment | 0.79 | 1.30 | 0.545 | -1.76 | 3.32 |
| Emotion regulation difficulties 1-month post-treatment <- Minimization response post-treatment | 0.82 | 1.55 | 0.597 | -2.31 | 3.87 |
| Emotion regulation difficulties 1-month post-treatment <- Emotion regulation difficulties pre-treatment | 0.97 | 0.10 | <0.001 | 0.77 | 1.15 |
| Emotion regulation difficulties 1-month post-treatment <- Minimization response post-treatment X Treatment | -2.29 | 2.23 | 0.304 | -6.77 | 1.99 |
| Emotion regulation difficulties 1-month post-treatment <- Treatment | -5.31 | 2.04 | 0.009 | -9.37 | -1.38 |

Abbreviations: NSSI, nonsuicidal self-injury; SE, standard error

# Table S7

Estimates and confidence intervals for direct and indirect effects

| Effects | Lower .5% | Lower 2.5% | Lower 5% | Estimate | Upper 5% | Upper 2.5% | Upper .5% |
| --- | --- | --- | --- | --- | --- | --- | --- |
| **Effects from Treatment to NSSI absence 1-month post-treatment** | | | | | | | |
| Total natural IE | -0.05 | -0.03 | -0.02 | 0.04 | 0.11 | 0.12 | 0.16 |
| Pure natural DE | -0.48 | -0.44 | -0.41 | -0.26 | -0.12 | -0.09 | -0.04 |
| Total effect | -0.42 | -0.38 | -0.36 | -0.22 | -0.08 | -0.06 | -0.01 |
| **Effects from Treatment to Emotion regulation difficulties 1-month post-treatment** | | | | | | | |
| Total natural IE | -1.49 | -1.01 | -0.71 | 0.56 | 2.04 | 2.46 | 3.38 |
| Pure natural DE | -11.15 | -9.86 | -9.25 | -5.75 | -2.44 | -1.78 | -0.43 |
| Total effect | -10.43 | -9.22 | -8.56 | -5.19 | -1.78 | -1.17 | -0.07 |

Abbreviations: NSSI, nonsuicidal self-injury; IE, indirect effect; DE, direct effect

# References

1. Bjureberg J, Ojala O, Hesser H, Häbel H, Sahlin H, Gratz KL, et al. Effect of Internet-Delivered Emotion Regulation Individual Therapy for Adolescents With Nonsuicidal Self-Injury Disorder: A Randomized Clinical Trial. JAMA Network Open. 2023 Jul 13;6(7):e2322069–e2322069.

2. Bjureberg J, Sahlin H, Hedman-Lagerlöf E, Gratz KL, Tull MT, Jokinen J, et al. Extending research on Emotion Regulation Individual Therapy for Adolescents (ERITA) with nonsuicidal self-injury disorder: open pilot trial and mediation analysis of a novel online version. BMC Psychiatry. 2018 Oct 11;18(1):326.

3. Hallquist MN, Wiley JF. MplusAutomation: An R Package for Facilitating Large-Scale Latent Variable Analyses in Mplus. Structural Equation Modeling: A Multidisciplinary Journal. 2018 Jul 4;25(4):621–38.

4. Epskamp S. semPlot: Path Diagrams and Visual Analysis of Various SEM Packages’ Output. R package version 1.1.2. [Internet]. 2019. Available from: https://CRAN.R-project.org/package=semPlot

5. Wickham H. Data Analysis. In: Wickham H, editor. ggplot2: Elegant Graphics for Data Analysis [Internet]. Cham: Springer International Publishing; 2016 [cited 2024 Aug 2]. p. 189–201. Available from: https://doi.org/10.1007/978-3-319-24277-4_9

6. R Core Team. R: A Language and Environment for Statistical Computing [Internet]. Vienna, Austria: R Foundation for Statistical Computing; 2023. Available from: https://www.R-project.org/

7. Rubin, D. B. Multiple imputation for nonresponse in surveys. 1987. Hoboken, NJ: Wiley
